# *Kabasura kudineer choornam*, a medicinal polyherbal formulation, modulates human macrophage polarization and phagocytic function

**DOI:** 10.55730/1300-0152.2752

**Published:** 2025-05-13

**Authors:** Aslı KORKMAZ, Duygu ÜNÜVAR, Sinem GÜNALP, Derya Göksu HELVACI, Duygu SAĞ

**Affiliations:** 1 Izmir Biomedicine and Genome Center, İzmir, Turkiye; 2 Department of Genomic Sciences and Molecular Biotechnology, Izmir International Biomedicine and Genome Institute, Dokuz Eylul University, İzmir, Turkiye; 3 Faculty of Medicine, Dokuz Eylul University, İzmir, Turkiye; 4 Department of Medical Biology, Faculty of Medicine, Dokuz Eylul University, İzmir, Turkiye

**Keywords:** Medicinal plants, polyherbal formulations, inflammation, viral infections, macrophage polarization

## Abstract

**Background/aim:**

*Kabasura kudineer choornam (KKC)* is a polyherbal formulation of 15 ingredients. It has antiinflammatory and antimicrobial properties and are effective in managing the symptoms of H1N1 swine flu and COVID-19. However, its mechanism of action is not fully understood. In this study, we examined the effect of *KKC* on the polarization and function of primary human macrophages.

**Materials and methods:**

Human monocyte-derived macrophages (M0 macrophages) pretreated with *KKC* extract were polarized into M1, M2a, or M2c subtypes. The expression of the M1/M2 polarization markers was analyzed using qPCR, flow cytometry, and ELISA, and the phagocytosis capacity of macrophages was analyzed using flow cytometry.

**Results:**

Our data show that the *KKC* treatment increased the expression of the M1 markers *IDO1*, *IL-1β*, *IL-12a* (p35), and *TNF* in both polarized and unpolarized macrophages at mRNA level. However, it decreased the secretion of IL-12 (p70) in M1 macrophages and increased the secretion of TNF in M0, M2a, and M2c macrophages. IL-10 secretion was increased in M0 and M2a macrophages, while it was decreased in M1 macrophages after the *KKC* treatment. Interestingly, all *KKC*-treated macrophage phenotypes displayed a downregulation in the expression of the M1/M2 surface markers CD64, CD206, CD209, and CD163, which also play a role in phagocytosis. In accordance with this result, the phagocytic capacity of both polarized and unpolarized macrophages was decreased after the *KKC* treatment.

**Conclusion:**

*KKC* extract modulates macrophage inflammatory response and could be a potential supplement for the treatment of infectious and inflammatory diseases.

## 1. Introduction

Studies have shown the preventive and therapeutic benefits of plants and their compounds in addressing common and complex diseases including cancer and various infectious diseases (Mahima et al., 2012; Dhama et al., 2013; Dhama et al., 2014a, 2014b; Rahal et al., 2014; Dhama et al., 2015; Mahesh et al., 2015; Singh et al., 2016). *Kabasura kudineer choornam (KKC)* is a polyherbal formulation derived from Siddha medicine originating in India (Sathiyarajeswaran et al., 2021). As shown in Table, the *KKC* formulation contains 15 different components (John et al., 2015; Mekala and Murthy, 2020; Behera et al., 2023) and is commonly recommended for the efficient management of prevalent respiratory conditions including colds, coughs, respiratory distress, and influenza-like symptoms (John et al., 2015; Mekala and Murthy, 2020). *KKC* has antiinflammatory, antiviral, antipyretic, immunomodulatory, and antibacterial properties due to its phytochemical components (Thillaivanan et al., 2015; Saravanan et al., 2018; Sathiyarajeswaran et al., 2021). It has been prescribed against influenza A virus subtype H1N1 (swine flu) and has shown great efficacy in managing disease symptoms and enhancing the immunological response of the body (Thillaivanan et al., 2015).

After the recent coronavirus disease 2019 (COVID-19) outbreak, in silico screening methods and molecular docking studies have indicated that the parent compounds of *KKC* may have antiviral effects against severe acute respiratory syndrome coronavirus 2 (SARS-CoV-2) by blocking the host cell receptor angiotensin converting enzyme 2 (ACE2) or inhibiting the key viral protease necessary for its replication in the host cell (Kiran et al., 2020). This action could potentially inhibit COVID-19 more effectively than synthetic drugs due to a superior energy score (Kiran et al., 2020; Vincent et al., 2020; Nallusamy et al., 2021). Different randomized clinical trials on COVID-19 patients have shown that *kabasura kudineer* as an adjunct treatment reduced SARS-CoV-2 viral burden, prevented progression from an asymptomatic to symptomatic state, improved symptoms, led to changes in immune markers, and decreased hospitalization length in COVID-19 patients without serious adverse events and mortality (Jamuna et al., 2021; Rao et al., 2020; Chitra et al., 2021; Meenakumari et al., 2021; Natarajan et al., 2021; Srivastava et al., 2021b). As a result, *KKC* is recommended as a therapeutic medicine for enhancing the immune system and combating COVID-19, as per the Ministry of Ayurveda, Yoga, Naturopathy, Unani, Siddha, and Homeopathy (AYUSH) guidelines issued by the Government of India (AYUSH Ministry of Health Corona Advisory—D.O. No. S. 16030/18/2019—NAM, March 6, 2020) (Jose et al., 2022).

Macrophages are innate immune cells of the myeloid lineage. They originate from blood monocytes that differentiate into either proinflammatory M1 (classically activated) or antiinflammatory M2 (alternatively activated) macrophages in presence of certain polarization factors when recruited into peripheral tissue (Gordon and Taylor, 2005; Davis et al., 2013; Italiani and Boraschi, 2014; Yang et al., 2014). Macrophages can be polarized into the M1 phenotype by IFNγ/LPS or TNF in vitro (Mantovani et al., 2005). M2 macrophages are categorized into 3 subgroups: M2a, M2b, and M2c, depending on the stimuli they respond to and the markers they express (Murray et al., 2014). M2a polarization is induced by IL-4 and/or IL13. M2b polarization is induced by immune complex and FcϒR/TLR ligands. M2c polarization is induced by IL-10, TGFβ, or glucocorticoid stimulation (Gordon, 2003). M1 macrophages support the antimicrobial and antitumoral response, while M2 macrophages are involved in tissue healing, allergic reactions, or cell proliferation depending on their subgroup (Munder et al., 1998; Mantovani et al., 2004). Imbalance between M1 and M2 macrophage phenotypes underlies numerous diseases. For instance, M1 macrophages in type 2 diabetes and atherosclerosis promote inflammation, obesity, and insulin resistance, while M2-like macrophages alleviate these symptoms in symptomatic plaques (Lumeng et al., 2007; Espinoza-Jiménez et al., 2012; Menghini et al., 2012; de Gaetano et al., 2016). Moreover, M1 macrophages have been linked to a strong type I interferon response and elevated disease severity and mortality in infections caused by viruses like SARS-CoV-2 (Feng et al., 2020; Mehta et al., 2020; Merad and Martin, 2020; Ziegler et al., 2020; Lv et al., 2021) and the highly virulent H5N1 influenza A virus (Baskin et al., 2009; Channappanavar et al., 2016).

Although *KKC* is known to enhance the immune response of the body, its mechanism of action is not well understood. In this study, we investigated the effect of *KKC* on the polarization and function of human macrophages. Our results show that *KKC* modulates polarization and decreases the phagocytic function of primary human macrophages with a downregulation of certain phagocytic surface receptors.

## 2. Materials and methods

### 2.1. Study approval

Buffy coats from healthy donors were obtained from Dokuz Eylül University Hospital blood bank (İzmir, Türkiye) after written consent. Ethical approval was granted by the Non-Interventional Research Ethics Committee of İzmir Biomedicine and Genome Center (İzmir, Türkiye) for the use of buffy coats (approval number: 2021-042).

### 2.2. Primary human monocyte isolation and macrophage differentiation

Primary human monocytes were isolated from buffy coats by gradient centrifugation using Ficoll-Paque (GE Healthcare, Pittsburgh, PA) followed by Percoll (GE Healthcare, Pittsburgh, PA) (Menck et al., 2014). Isolated primary monocytes were then cultured in RPMI-1640 medium (Gibco, ThermoFisher Scientific, Waltham, MA, USA) completed with 5% heat-inactivated fetal bovine serum (FBS) (Gibco, ThermoFisher Scientific, Waltham, MA, USA) and 1% penicillin-streptomycin (Gibco, ThermoFisher Scientific, Waltham, MA, USA) (R5 medium), supplemented with 10 ng/mL recombinant human M-CSF (PeproTech, Rocky Hill, NJ) in ultralow attachment 6-well plates (Corning Life Sciences, Tewksbury, MA) for 7 days at 37 °C and 5% CO_2_ (Gunalp et al., 2023). After 7 days, the cells were collected, and the macrophages were verified to be approximately 90% CD68+ by flow cytometry.

### 2.3. Extraction and preparation of *KKC*

*KKC* in powder form was kindly provided by Dr Ravi Kumar Reddy from Sri Sri Tattva (Bengaluru, Karnataka, India). As *KKC* contains active compounds including alkaloids, carbohydrates, glycosides, flavonoids, phenols, saponins, tannins, and terpenoids and also in small quantities of phlobatannins, essential oils, vitamin C, proteins, and amino acids derived from diverse medicinal plants (Mekala and Murthy, 2020), we obtained *KKC* extract by dissolving the fine powder in DMSO. DMSO can dissolve polar and nonpolar compounds (Kuroda et al., 2020). The stock solution concentration was set to 100 mg/mL. Following 6 h of gentle agitation and 18 h of upright settling at room temperature, the solution was centrifuged at 200 × gravity for 5 min. The supernatants were kept at −80 °C for future use.

### 2.4. Treatments

Human monocyte-derived macrophages (MDMs) were plated into 24-well plates at 6.5 × 10^5^ cells/mL per well in R5 medium and rested overnight at 37 °C and 5% CO_2_. Naive macrophages (M0) were either pretreated with 500 μg/mL. *KKC* extract for 2 h, then polarized into M1 with 100 ng/mL LPS (Ultrapure, InvivoGen, San Diego, CA, USA) and 20 ng/mL IFNγ (R&D, Minneapolis, MN, USA), M2a with 20 ng/mL IL-4 (R&D, Minneapolis, MN, USA), M2c with 20 ng/mL IL-10 (R&D, Minneapolis, MN, USA) for 22 h, or left unstimulated in accordance with the literature (Wang et al., 2014; Iqbal and Kumar, 2015; Raggi et al., 2017; Purcu et al., 2022).

### 2.5. Flow cytometry

Single-cell suspensions were obtained by detaching the cells from the culture plates using StemPro Accutase Cell Dissociation Reagent (Gibco, Thermo Fisher Scientific, Waltham, MA, USA) according to the manufacturer’s instructions. Exclusion of dead cells and cell viability determination was performed using the Zombie UV fixable viability kit (BioLegend, San Diego, CA, USA). Surface antigen staining was performed in flow cytometry staining buffer (PBS supplemented with 1% bovine serum albumin, 0.1% sodium azide). Fcγ receptors were blocked prior to surface antigen staining by incubating the cells in flow cytometry staining buffer containing human TruStain FcX antibody (BioLegend, San Diego, CA, USA) for 15 min on ice. Surface staining was performed using the following fluorochrome labelled antibodies (BioLegend, San Diego, CA, USA) in flow cytometry staining buffer at given concentrations for 45 min on ice in the dark: CD86-BV605 (IT2.2, 1:200), CD38-BV510 (HB-7, 1:100), HLA-DR-APC-Cy7 (L243, 1:200), CD64-BV421 (OX-108, 1:200), CD206-AF700 (15.2, 1:200), CD209-PE (9E9A8, 1:200), CD200R-PE-Dazzle594 (OX108, 1:200), CD163-BV510 (GHI/61, 1:200). The cells were stained in v-bottom 96-well plates (Greiner Bio-One, NC, USA). Following the staining process, samples were washed, resuspended in flow cytometry buffer and transferred in FACS tubes (Greiner Bio-One, NC, USA). Fluorescence was detected using an LSRFortessa analyzer (BD Biosciences, USA). The data were analyzed with FlowJo software (TreeStar, Ashland, OR, USA).

### 2.6. Total RNA isolation and real-time PCR

Total RNA isolation of samples was performed using Monarch Total RNA Miniprep Kit (New England Biolabs, MA, USA) according to the manufacturer’s protocol. The purity and quantity of RNA were assessed using a Nanodrop spectrophotometer (Thermo Fisher Scientific, Waltham, MA, USA). For each sample, 1000 ng of RNA was converted to cDNA using EvoScript Universal cDNA Master (Roche, Basel, Switzerland) according to the manufacturer’s instructions. M1 and M2 polarization markers, Type I IFN, and housekeeping gene expression was assessed using Fast Start Essential DNA Green Master (Roche, Basel, Switzerland) and RealTime ready Single Assay Primers for *IDO1* (#QT00000504), *IL-1β* (QT00021385), *IL-12A* (QT00000357), *TNF* (QT00029162), *TGM2* (QT00081277), *MRC1* (CD206) (QT00012810), *CD163* (QT00074641), and *IL-10* (QT00041685). In accordance with the manufacturer’s instructions, the reaction mixture was prepared on ice with a final volume of 12.5 μL, containing 1.25 μL of 10× QuantiTect Primer Assay, 6.25 μL of 2× DNA Green master mix, 2.5 μL of RNase/DNase-free water, and 2.5 μL of diluted cDNA. Thermal cycling conditions were as follows: an initial enzyme activation step at 95 °C for 10 min, followed by 40 amplification cycles consisting of denaturation at 95 °C for 15 s, annealing at 60 °C for 20 s, and extension at 72 °C for 30 s. This was followed by a melting curve analysis, initiated at 95°C for 30 s, then incubation at 60 °C for 2 min, a gradual increase to 95 °C, and final cooling at 40 °C for 30 s to stabilize the instrument. All reactions were performed in technical triplicates. Real-time PCR analysis was performed using Applied Biosystems 7500 Fast Real-time PCR System (Thermo Fisher Scientific, Waltham, MA, USA). For normalization, the housekeeping gene *β*-actin (*ACTB*) (QT00095431) was used. The 2^−ΔΔCt^ method was used to calculate relative mRNA expression (Livak and Schmittgen, 2001).

### 2.7. ELISA

Supernatants of samples were collected after stimulation and stored at −20 °C until further usage. Concentrations of cytokines were determined using the following kits according to the manufacturer’s instructions: IL-12p70 (431704; BioLegend, San Diego, CA, USA), TNF (430204; BioLegend, San Diego, CA, USA), and IL-10 (430604; BioLegend, San Diego, CA, USA).

### 2.8. Phagocytosis assay

For the assessment of phagocytosis, pHrodo Red conjugated *Staphylococcus aureus* bioparticles (A10010, Invitrogen, Thermo Fisher Scientific, Waltham, MA, USA) was used. Macrophages were seeded in 24-well cell culture plates at 6.5 × 10^5^ cells/mL per well in R5 medium. After the stimulation times indicated above, supernatants were collected, cells were washed with PBS and detached from the culture plate using StemPro accutase cell dissociation reagent (Gibco, Thermo Fisher Scientific, Waltham, MA, USA) according to the manufacturer’s instructions. After washing, macrophages were incubated with pHrodo Red conjugated *S*. *aureus* at 37 °C (and 4 °C for control) for 2 h. Then, Zombie UV fixable viability kit (BioLegend, San Diego, CA, USA) staining was performed to eliminate dead cells, and phRodo Red fluorescence emission was detected by flow cytometry using an LSRFortessa analyzer (BD Biosciences, USA). The data was analyzed with the FlowJo software (TreeStar, Ashland, OR, USA). Doublets were excluded from the analysis using forward- and side-scatter parameters.

### 2.9. Statistical analyses

GraphPad Prism version 9 (GraphPad Software, CA, USA) was used to analyze descriptive statistics and to generate graphs. The Shapiro-Wilk normality test was used to determine whether the continuous variables were normally distributed. For normally distributed data analyses, 2-tailed Student’s t-test was used for 2 independent samples and one-way ANOVA was applied for comparison of more than 2 samples. A p-value less than 0.05 was considered statistically significant.

## 3. Results

### 3.1. The impact of the *KKC* extract on the viability of primary human macrophages

**Figure 1:**
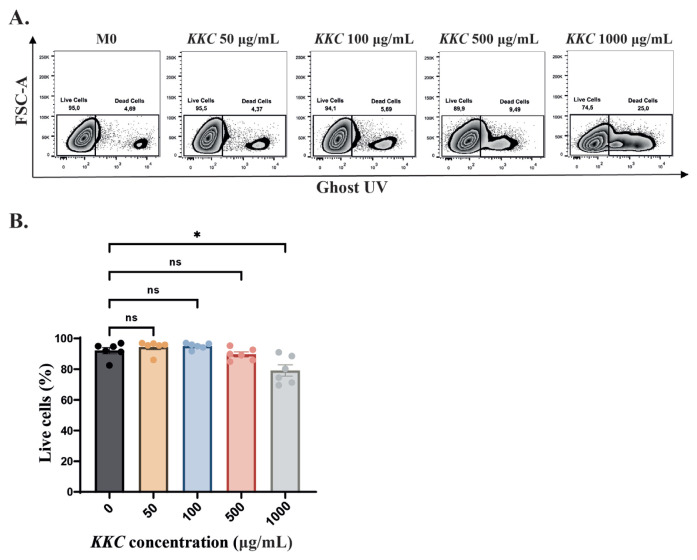
The effect of *KKC* extract on the viability of human MDMs. Unpolarized primary human MDMs (M0) were incubated for 24 h either without treatment (control) or with *KKC* extract at concentrations of 50, 100, 500, or 1000 μg/mL. Macrophages were stained with a Zombie UV fixable viability kit (Biolegend) and analyzed by flow cytometry. A) Representative dot blots, B) bar graphs showing the percentages of live cells. Data are shown in mean ± SEM of biological replicates of 6 donors (n = 6) pooled from 3 independent experiments. One-way ANOVA followed by Dunnet’s posthoc test was performed for the statistical analyses. *p < 0.05, ns = not significant.

First, we analyzed the effect of *KKC* on the viability of primary human macrophages. The cells were incubated for 24 h either without treatment (control) or with *KKC* extract at concentrations of 50, 100, 500, or 1000 μg/mL. The cells were stained with Ghost UV and analyzed by flow cytometry. Figure 1 shows that the cell viability was not significantly affected by *KKC* extract at the concentrations of 50, 100, and 500 μg/mL. Although treating the macrophages with 1000 μg/mL *KKC* decreased cell viability significantly compared to untreated control, more than 78% of the cells were viable.

### 3.2. The impact of the *KKC* extract on the expression of M1 and M2 markers in primary human macrophage phenotypes at mRNA level

**Figure 2:**
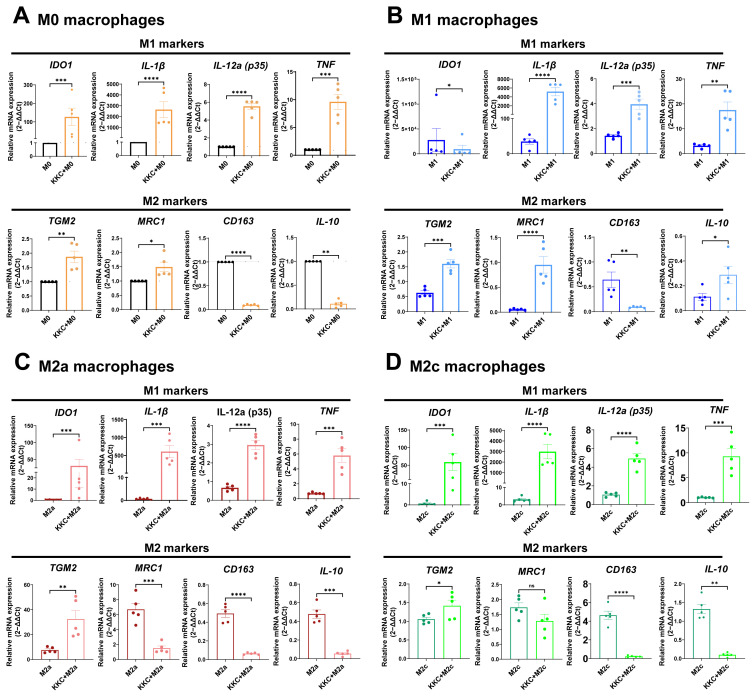
The effect of *KKC* extract on the expression of M1/M2 markers by unpolarized and polarized human MDMs at mRNA level. Unpolarized primary human MDMs were only treated with 500 μg/mL *KKC* extract (*KKC* + M0 group) for 24 h or treated with 500 μg/mL DMSO as a control (M0 group) for 24 h. For polarization, human MDMs were prestimulated with 500 μg/mL of *KKC* extract for 2 h and then polarized into M1 (100 ng/mL and 20 ng/mL IFNγ), M2a (20 ng/mL IL-4), and M2c (20 ng/mL IL-10) phenotypes for 22 h. Polarization controls were only stimulated with M1, M2a, or M2c agents and 500 μg/mL DMSO (for the experimental setup). qPCR analysis of common M1 and M2 markers in (A) M0, (B) M1, (C) M2a, and (D) M2c macrophages are shown. Data are shown in mean ± SEM of biological replicates of 5 donors (n = 5) pooled from 3 independent experiments. A 2-tailed paired Student’s t-test was performed for the statistical analyses comparing controls and *KKC* extract treated groups. *p < 0.05, **p < 0.01, ***p < 0.001, ****p < 0.0001.

Next, we examined how the *KKC* extract affected the expression of conventional M1 and M2 macrophage markers, which are widely accepted in the literature (Martinez et al., 2006; Tóth et al., 2009; Martinez and Gordon, 2014; Nadella et al., 2015; Rőszer, 2015). The relative mRNA levels of M1 genes *IL-1β*, *IL-12a*, and *TNF* increased in M0, M1, M2a, and M2c macrophages treated with 500 μg/mL *KKC* extract (Figure 2). *IDO1* also increased in macrophage subtypes except in M1 macrophages (Figure 2). In contrast, the relative expression levels of M2 genes *CD163* and *IL-10* decreased in *KKC*-treated M0, M2a, and M2c macrophages (Figures 2A, 2C, and 2D). In M1 macrophages, while *CD163* expression decreased, *IL-10* expression increased (Figure 2B). Moreover, contrary to expectations, *TGM2* expression increased in *KKC*-treated M0, M1, M2a, and M2c macrophages (Figures 2A–2D). After treatment with the *KKC* extract, while the expression level of another M2 marker *MRC1* increased in M0 and M1 macrophages (Figures 2A–2B), it decreased in M2a and M2c macrophages (Figures 2C and 2D). Our data show that the *KKC* extract increased the expression of M1 markers in unpolarized and polarized macrophages at the mRNA level. However, *KKC* increased or decreased gene expression depending on the M2 markers and the macrophage subtypes.

### 3.3. The impact of the *KKC* extract on the expression of M1 and M2 markers in primary human macrophage phenotypes at the protein level

**Figure 3:**
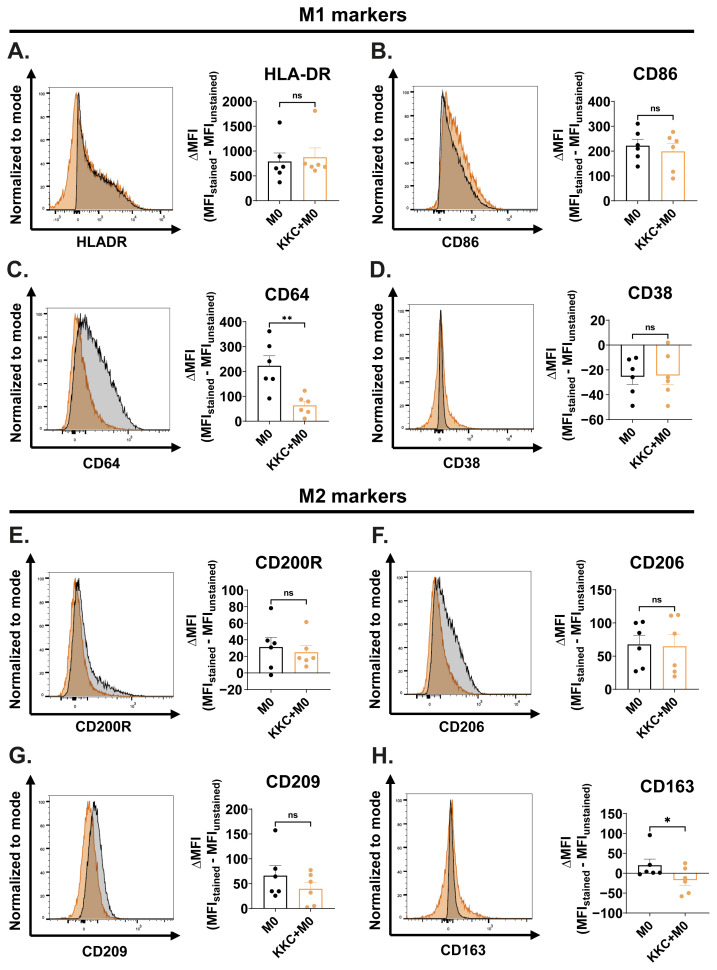
The effect of *KKC* extract stimulation on the expression of M1/M2 surface markers in M0 macrophages. Unpolarized primary human MDMs were only treated with 500 μg/mL *KKC* extract (*KKC*+M0 group) 500 μg/mL DMSO as a control (M0 group) for 24 h. (A–D) Expression of HLA-DR, CD86, CD64, and CD38 M1 markers, (E–H) CD200R, CD206, CD209, and CD163 M2 markers were analyzed by flow cytometry, and the representative plots are included. Data are shown as mean ± SEM of biological replicates of 6 donors (n = 6) pooled from 3 independent experiments. A 2-tailed paired Student’s t-test was performed for the statistical analyses comparing controls and *KKC* extract treated groups. *p < 0.05, **p < 0.01, ***p < 0.001, ****p < 0.0001, ns = not significant. MFI: median fluorescence intensity.

Next, we examined the impact of the *KKC* extract on the expression of conventional M1 and M2 macrophage markers (Gordon, 2003; Italiani and Boraschi, 2014; Martinez and Gordon, 2014; Murray et al., 2014) at the protein level. Figure 3 shows the effect of the *KKC* extract stimulation on the expression of M1/M2 surface markers in M0 macrophages. *KKC* treatment did not affect the expression of M1 markers HLA-DR, CD86, and CD38; and M2 markers CD200R, CD206, and CD209 (Figures 3A, 3B, and 3D–G). However, a statistically significant decrease in the expression of the M1 marker CD64 and M2 marker CD163 was observed after *KKC* treatment compared to control (Figures 3C–3H).

**Figure 4:**
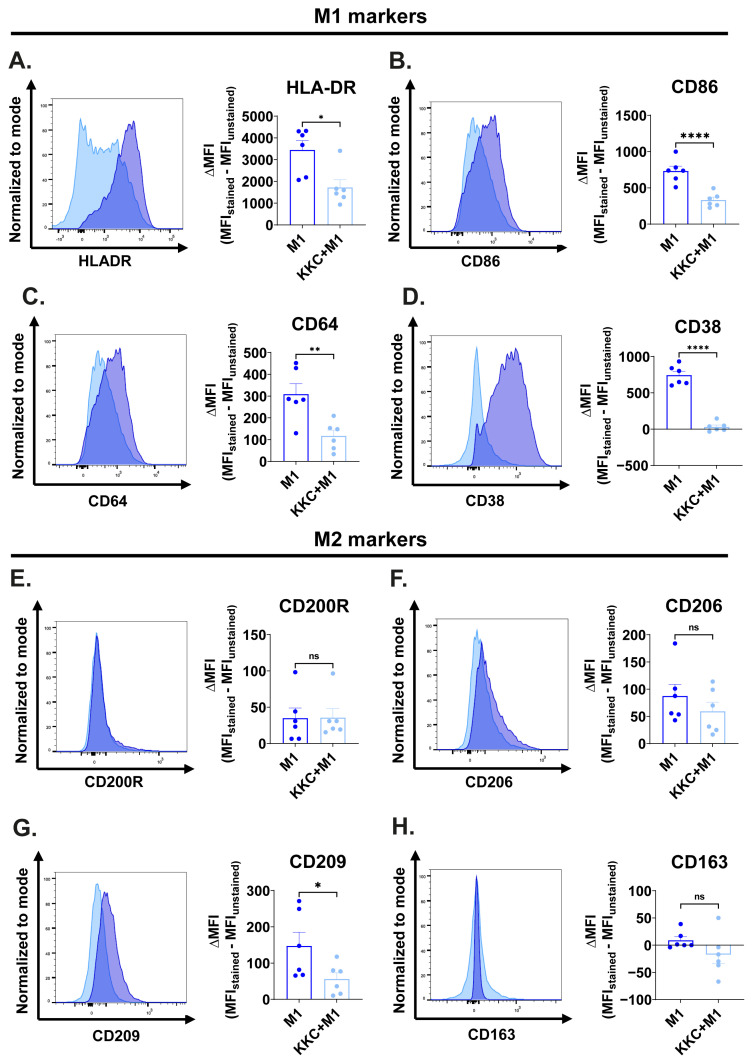
The effect of *KKC* extract stimulation on the expression of M1/M2 surface markers in M1 macrophages. Primary human macrophages were prestimulated with 500 μg/mL *KKC* extract for 2 h and then polarized into M1 (100 ng/mL LPS + 20 ng/mL IFNγ) macrophages (*KKC* +M1 group) for 22 h. Polarization controls were treated with 500 μg/mL DMSO for 2 h and with M1 agents for 22 h (M1 groups). (A–D) Expression of HLA-DR, CD86, CD64, and CD38 M1 markers and (E–H) CD200R, CD206, CD209, and CD163 M2 markers were analyzed by flow cytometry, and the representative plots are included. Data are shown as mean ± SEM of biological replicates of 6 donors (n = 6) pooled from 3 independent experiments. A 2-tailed paired Student’s t-test was performed for the statistical analyses comparing controls and *KKC* extract treated groups. *p < 0.05, **p < 0.01, ***p < 0.001, ****p < 0.0001, ns = not significant. MFI: median fluorescence intensity.

The impact of *KKC* extract stimulation on M1/M2 surface marker expression in M1 macrophages is shown in Figure 4. M1 macrophages showed a significant decrease in the expression of the M1 markers HLA-DR, CD86, CD64, and CD38; and M2 marker CD209 after *KKC* stimulation compared to the control (Figures 4A–D and 4G). Nevertheless, the expression levels of M2 markers CD200R, CD206, and CD163 did not change in M1 macrophages after treatment with the *KKC* extract (Figures 4E, 4F, and 4H).

**Figure 5:**
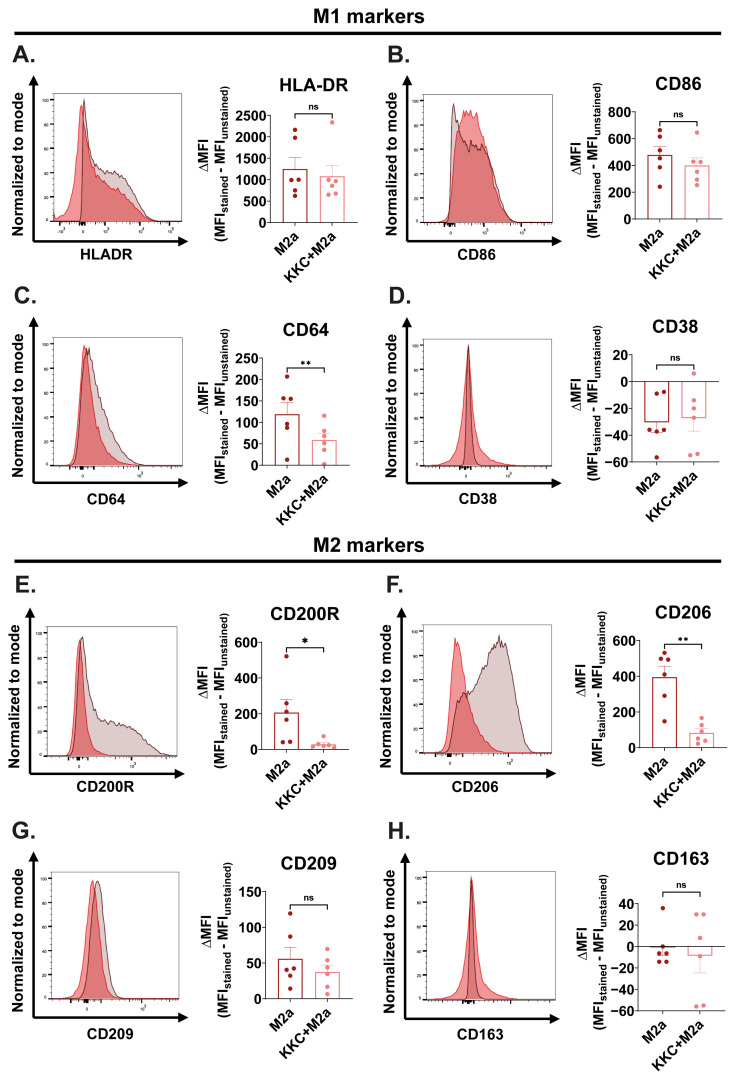
The effect of *KKC* extract stimulation on the expression of M1/M2 surface markers in M2a macrophages. Primary human macrophages were prestimulated with 500 μg/mL *KKC* extract for 2 h and then polarized into M2a (20 ng/mL IL-4) macrophages (*KKC*+M2a group) for 22 h. Polarization controls were treated with 500 μg/mL DMSO for 2 h and with M2a agent for 22 h (M2a group). (A–D) Expression of HLA-DR, CD86, CD64, and CD38 M1 markers and (E–H) CD200R, CD206, CD209, and CD163 M2 markers were analyzed by flow cytometry, and the representative plots are included. Data are shown as mean ± SEM of biological replicates of 6 donors (n = 6) pooled from 3 independent experiments. A 2-tailed paired Student’s t-test was performed for the statistical analyses comparing controls and *KKC* extract treated groups. *p < 0.05, **p < 0.01, ***p < 0.001, ****p < 0.0001, ns = not significant. MFI: median fluorescence intensity.

Figure 5 shows the effect of the *KKC* extract stimulation on M1/M2 surface marker expression in M2a macrophages. *KKC* treatment did not significantly affect the expression of the M1 markers HLA-DR, CD86, and CD38; and M2 markers CD209 and CD163 (Figures 5A, 5B, 5D, 5G, and 5H). However, a statistically significant decrease in the expression of M1 marker CD64; and M2 markers CD206 and CD200R (Figures 5C, 5E, and 5F) was observed in *KKC*-treated M2a macrophages compared to M2a control macrophages.

**Figure 6:**
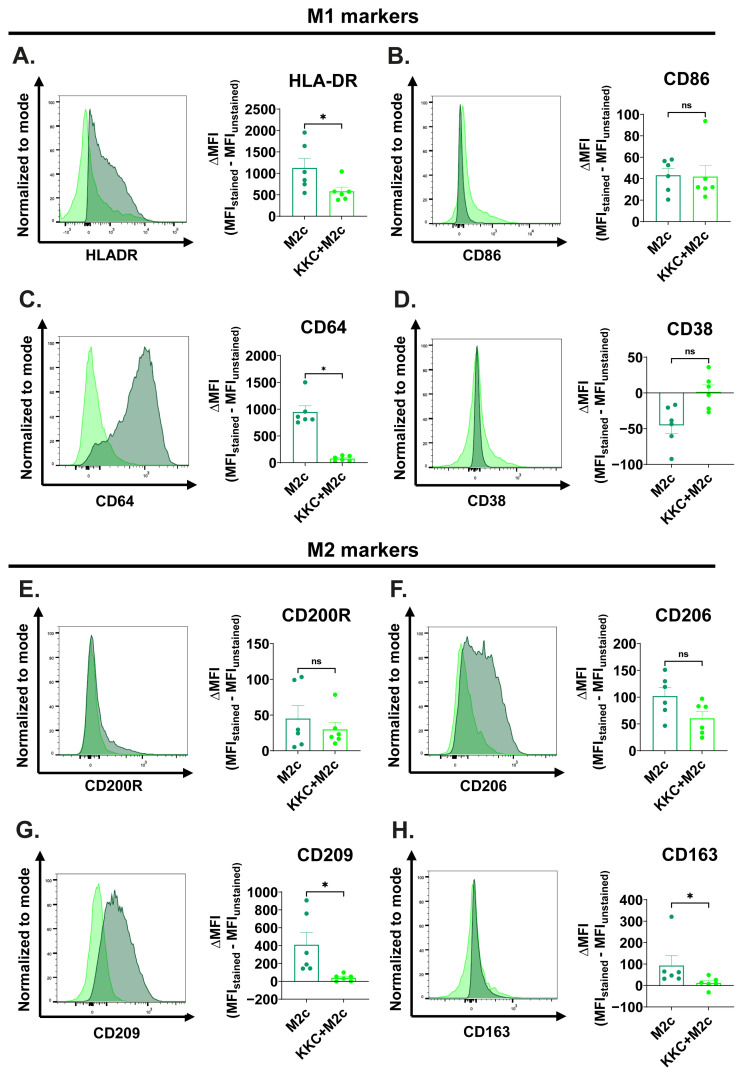
The effect of *KKC* extract stimulation on the expression of M1/M2 surface markers in M2c macrophages. Primary human macrophages were prestimulated with 500 μg/mL *KKC* extract for 2 h and then polarized into M2c (20 ng/mL IL-10) macrophages (*KKC* +M2c group) for 22 h. Polarization controls were treated with 500 μg/mL DMSO for 2 h and with M2c agent for 22 h (M2c group). (A–D) Expression of HLA-DR, CD86, CD64, and CD38 M1 markers and (E–H) CD200R, CD206, CD209, and CD163 M2 markers were analyzed by flow cytometry, and the representative plots are included. Data are shown as mean ± SEM of biological replicates of 6 donors (n = 6) pooled from 3 independent experiments. A 2-tailed paired Student’s t-test was performed for the statistical analyses comparing controls and *KKC* extract treated groups. *p < 0.05, **p < 0.01, ***p < 0.001, ****p < 0.0001, ns = not significant. MFI: median fluorescence intensity.

Figure 6 shows the effect of the *KKC* extract stimulation on the expression of the M1/M2 surface markers in M2c macrophages. After the *KKC* treatment, a statistically significant reduction in the expression of the M1 markers HLA-DR and CD64; and M2 markers CD209 and CD163 was observed (Figures 6A, 6C, 6G, and 6H) in M2c macrophages compared to the untreated control. *KKC* treatment did not significantly affect the expression of the M1 markers CD86 and CD38, and M2 markers CD200R and CD206 (Figures 6B, 6D, 6E, and 6F).

**Figure 7:**
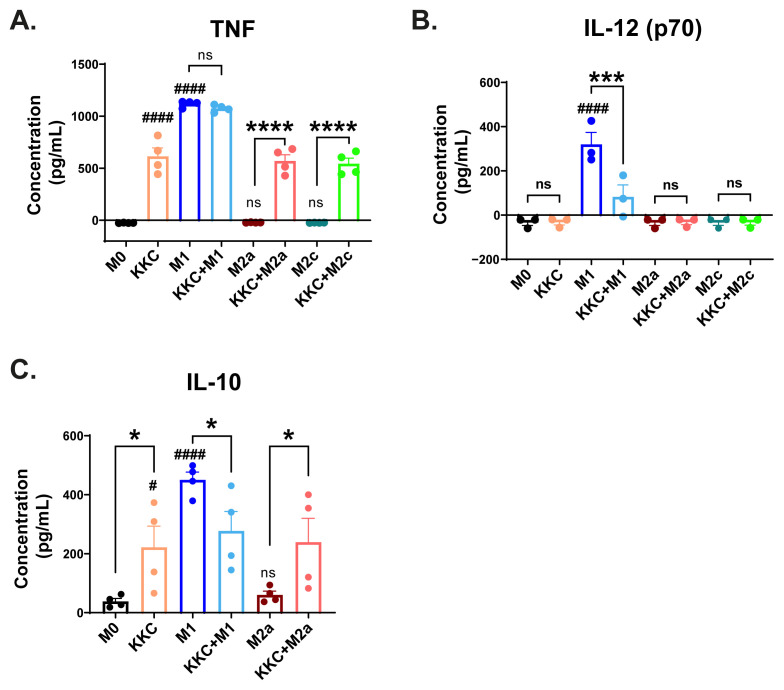
The effect of *KKC* extract on M1/M2 cytokine production by polarized and unpolarized MDMs. Unpolarized primary human MDMs were only treated with 500 μg/mL *KKC* extract (*KKC*+M0 group) for 24 h. For polarization, human MDMs were prestimulated with 500 μg/mL *KKC* extract for 2 h and then polarized into M1 (100 ng/mL and 20 ng/mL IFNγ), M2a (20 ng/mL IL-4) and M2c (20 ng/mL IL-10) for 22 h (M1, M2a, or M2c +*KKC* groups). Polarization controls were only stimulated with M1, M2a, or M2c agents (M1, M2a, or M2c groups). ELISA analysis of (A) TNF (B) IL-12 (p70) and (C) IL-10 are shown. Data are shown as mean ± SEM of biological replicates of 6 donors (n = 6) pooled from 3 independent experiments. One-Way ANOVA followed by Sidak’s posthoc test was performed for the statistical analyses comparing controls and *KKC* extract treated groups. # indicates p-values obtained by comparing M1, M2a, and M2c with M0 control. * indicates p-values obtained by comparing M1 vs. *KKC*+M1, M2a vs. *KKC*+M2a, and M2c vs. *KKC*+M2c. *, # p < 0.05, **, ## p < 0.01, ***, ### p < 0.001, ****, #### p < 0.0001, ns = not significant.

Next, we measured the levels of M1 cytokines TNF and IL-12, as well as M2 cytokine IL-10 in M0, M1, M2a, and M2c macrophages using ELISA. As shown in Figure 7, the *KKC* extract treatment increased the production of TNF in M0, M2a, and M2c macrophages, and it did not further increase the production of TNF by M1 macrophages (Figure 7A). There was no IL-12 (p70) production in M0, M2a, and M2c macrophages. However, there was IL-12 (p70) production in M1 macrophages, and the level decreased after *KKC* extract treatment compared to untreated M1 macrophages (Figure 7B). Interestingly, *IL-10* showed a decrease in mRNA expression (Figures 2A–2C) but an increase in secretion in M0 and M2a macrophages (Figure 7C), while the opposite response was observed in M1 macrophages (Figures 2B–2D, 7C).

Overall, our data suggest that *KKC* extract increases the levels of some M1 markers but mostly acts on the modulation of the macrophage inflammatory response.

### 3.4. The impact of the *KKC* extract on the phagocytosis capacity of primary human macrophage phenotypes

**Figure 8:**
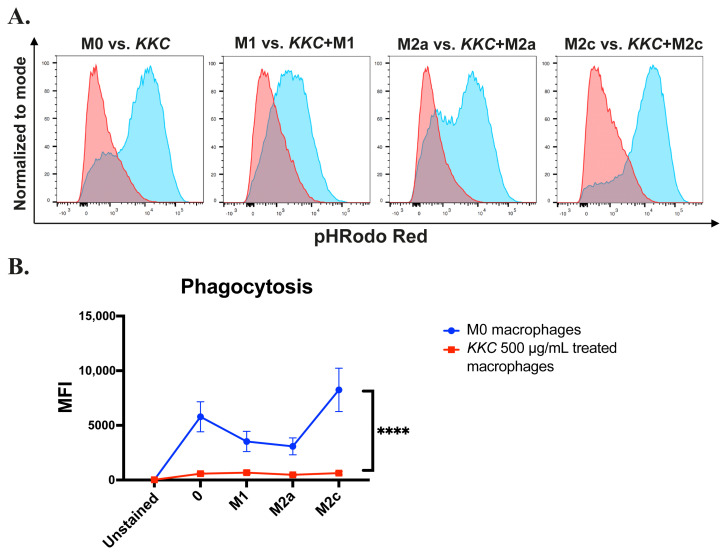
The effect of *KKC* extract on the phagocytic activity of polarized and unpolarized human MDMs. Unpolarized primary human MDMs were only treated with 500 μg/mL *KKC* extract for 24 h (*KKC*+M0 group). For polarization, human MDMs were prestimulated with 500 μg/mL *KKC* extract for 2 h and then polarized into M1 (100 ng/mL and 20 ng/mL IFNγ), M2a (20 ng/mL IL-4), and M2c (20 ng/mL IL-10) for 22 h (M1, M2a, or M2c +*KKC* groups). Polarization controls were only stimulated with M1, M2a, or M2c agents (M1, M2a, or M2c groups). Macrophages were incubated with pHrodo Red-tagged *S*. *aureus* bioparticle for 2 h at 37°C. Fluorescence in the PE-channel was analyzed by flow cytometry. A) Representative histograms, B) line graphs showing the pHRodo Red positive mean fluorescence intensities (MFIs). Data are shown as mean ± SEM of biological replicates of 6 donors (n = 6) pooled from 3 independent experiments. A 2-way ANOVA was performed for the statistical analyses comparing controls and *KKC* extract treated groups. *p < 0.05, **p < 0.01, ***p < 0.001, ****p < 0.0001.

During our investigation into the effect of *KKC* extract on macrophage polarization, we discovered a decrease in the protein expression levels of surface markers CD64, CD206, CD209, and CD163 in the M0, M1, M2a, and M2c macrophage groups (Figures 2–6). While these markers are commonly linked to the polarization of macrophages, they also have a significant impact on the phagocytic activity of these cells (Fabriek et al., 2005; McGreal et al., 2005; Martinez et al., 2008; Etzerodt and Moestrup, 2013; Azad et al., 2014; Bournazos et al., 2016; Akinrinmade et al., 2017). Hence, the effect of the *KKC* extract on the phagocytic capacity of human MDMs was analyzed using pHRodo conjugated heat-killed *S*. *aureus* bioparticle. Our data showed that the *KKC* extract prominently decreased the phagocytic capacity of unpolarized and polarized macrophages (Figure 8).

## 4. Discussion

In the last 20 years, medicinal plant research become more important. Many studies have shown that plants and their chemicals can prevent and treat a variety of diseases including cancer and influenza. The polyherbal formulation *KKC*, comprised of 15 ingredients (John et al., 2015; Mekala and Murthy, 2020; Behera et al., 2023), has been shown to have antiinflammatory, antipyretic, and antibacterial activities (John et al., 2015; Thillaivanan et al., 2015; Mekala and Murthy, 2020; Natarajan et al., 2021). In addition, in in silico studies, *KKC* has high binding affinity and interactions with the SARS-CoV-2 spike protein (Kiran et al., 2020; Kumar et al., 2020; Vincent et al., 2020; Maideen, 2021; Nallusamy et al., 2021) and a few clinical studies have shown *KKC* has effective antiviral properties against SARS-CoV-2 (Natarajan et al., 2021; Meenakumari et al., 2021; Srivastava et al., 2021a, 2021b). Following these promising results, a limited number of studies have investigated the effects and mechanism of action of *KKC* on the immune response. Jose et al. (2022) have shown that the aqueous extract of *KKC* has an antioxidant and antiinflammatory effect by inhibiting the production of intracellular ROS and NO and decreasing the production of proinflammatory cytokines in a mouse macrophage cell line. Furthermore, Behera et al. (2023) investigated the effect of *KKC* on immunomodulation using Jurkat T cells. They found that *KKC* increased the expression of antiinflammatory IL-10Rα and IL-10 cytokines in LPS induced T cells, indicating a potential role in immunomodulation. To the best of our knowledge, there is currently no other studies that examine the effect of *KKC* on macrophage polarization.

In the presence of certain polarization factors, macrophages can be polarized into proinflammatory M1 macrophages and antiinflammatory M2 macrophages (Italiani and Boraschi, 2014; Yang et al., 2014). A disrupted balance between these phenotypes can progress cancer, infectious, and inflammatory diseases (Espinoza-Jiménez et al., 2012; Menghini et al., 2012; Kraakman et al., 2014; Murray et al., 2014). M1 macrophages have been linked to a strong type I interferon response and elevated disease severity and mortality in infections caused by viruses like SARS-CoV-2 (Feng et al., 2020; Mehta et al., 2020; Merad and Martin, 2020; Ziegler et al., 2020; Lv et al., 2021) and the highly virulent H5N1 influenza A virus (Baskin et al., 2009; Channappanavar et al., 2016). In such infections, the severity of the disease is determined by the severity of the so called “cytokine storm,” caused by the overproduction of proinflammatory mediators and the overstimulation of the inflammatory response. Macrophages play a significant role in this process (Jafarzadeh et al., 2020; Kosyreva et al., 2021). Achieving a balanced activation of the M2-like phenotype is crucial to restrict the immunopathological response caused by the infection (Shirey et al., 2014; Sang et al., 2015). Therefore, identifying modulators regulating macrophage polarization is crucial for effective immunotherapies. The effect of *KKC* on macrophage polarization is not known. Here, we report a detailed analysis of the impact of *KKC* on the polarization and function of primary human MDMs.

To determine the effect of the *KKC* extract on human macrophage polarization, we first analyzed the conventional polarization markers at the mRNA level. We found that although the expression of the M1 markers increased in unpolarized M0, M1, M2a, and M2c polarized macrophages, the expression of the M2 marker TGM2 also increased (Figure 2). Although *TGM2* is widely accepted as an M2 marker (Martinez et al., 2013), there are contradictory studies suggesting that *TGM2* expression may be induced in some inflammatory conditions (Kim et al., 2006; Verma and Mehta, 2007; Sun and Kaartinen, 2018). *TGM2* could also activate TGFβ that in turn induces profibrotic and antiinflammatory cytokines (Mehta et al., 2010). More importantly, TGM2 plays a crucial function in efferocytosis (Eligini et al., 2016), a process essential for tissue repair, inflammation resolution, and maintaining immune system balance in homeostasis (Ge et al., 2022). Thus, it can be inferred that the induction of TGM2 contributes to the immunomodulatory effect of *KKC* (Figure 2). Moreover, *IL-10* gene expression decreased in unpolarized M0, M2a, and M2c polarized macrophages, but increased in M1 polarized macrophages at the mRNA level (Figure 2). This result partially aligned with the literature (Behera et al., 2023).

Interestingly, an opposite effect was observed at the protein level. The secretion of IL-10 increased in M0 and M2a macrophages, while it decreased in M1 macrophages after the *KKC* treatment (Figure 7). Gene expression at the transcriptional and translational levels may be inconsistent depending on differences between individuals, different genes, and their biological category, as seen in previous genome-wide studies in human liver and lung tissues, as well as in yeast (Guo et al., 2008; Kalb et al., 2019). These inconsistencies may be due to transcriptional and posttranscriptional splicing, translational modifications, translational regulation, and protein complex formations. Furthermore, both biological and experiment-specific variations in mRNA and protein degradation rates may further affect the strength of the correlation between mRNA and protein expression (Guo et al., 2008). Either way, IL-10 can still be considered as a part of the immunomodulatory effect of *KKC*, and further investigation is needed.

Macrophages are professional phagocytes (Aderem and Underhill, 1999). They express a variety of receptors to facilitate this function (Hirayama et al., 2017). M1 and M2 macrophages also differ in their phagocytic activity (Leidi et al., 2009). M2 macrophages have higher phagocytic capacity than M1 macrophages (Gratchev et al., 2005; Lingnau et al., 2007). However, this is highly dependent on the expressed surface markers such as CD206, CD163, and CD209 (Schulz et al., 2019) and the environmental conditions. For instance, the Fc gamma receptor FcγRI (CD64) has been reported as an M1 polarization marker in the literature (Murray et al., 2014) while having a fundamental role in receptor-mediated phagocytosis. Another M1 marker CD38, found to be selectively upregulated after IFNγ treatment (Schulz et al., 2019), is also associated with phagocytosis capacity due to its function in releasing Ca^2+^ ions, which could enhance the process of particle engulfment in a manner comparable to Fcγ receptor-mediated phagocytosis (Kang et al., 2012). The CD206 marker is recognized as an M2a polarization marker in the literature (Martinez et al., 2008). This mannose scavenger receptor is also involved in phagocytosis (Azad et al., 2014; Schulz et al., 2019; Xu et al., 2019). CD163 has been identified as an M2c marker (Mantovani et al., 2013; Shapouri-Moghaddam et al., 2018; Wang et al., 2020) and its expression also renders macrophage phagocytosis competent (Fabriek et al., 2005; Schulz et al., 2019). CD209 (DC-SIGN) is a C-type lectin receptor that recognizes high mannose-type N-glycans, binds with high affinity (Geurtsen et al., 2010), and is involved in phagocytosis (McGreal et al., 2005). Schulz et al. (2019) discovered that CD209 is directly associated with the amount of particle uptake during phagocytosis. Importantly, studies have shown that CD209 can directly bind to the S glycoprotein of SARS-CoV-2 and cooperate with ACE2 for viral entry of SARS-CoV-1 via endocytosis (Yang et al., 2004; Han et al., 2007; Xu et al., 2020). High expression of CD209 also triggers an immune response and can lead to a severe response to SARS-CoV-2 infection in cancer patients (Li et al., 2022). When the effect of *KKC* extract on M1/M2 macrophage polarization in primary human macrophages was examined at the protein level, a decrease in the expression of phagocytic surface markers CD64, CD206, CD209, and CD163 was observed in M0, M1, M2a, and M2c macrophage groups (Figures 3–6). Downregulation of another phagocytic surface marker CD38 was also observed in M1 macrophages. (Figure 3).

The changes observed in the surface marker expression levels led us to investigate the phagocytosis function of polarized and unpolarized macrophages after *KKC* stimulation. As shown in the Figure 8, overall phagocytosis capacity of human MDMs was prominently decreased after the *KKC* treatment, as expected. In infections caused by viruses such as SARS-CoV-1, SARS-CoV-2 (Feng et al., 2020; Mehta et al., 2020; Merad and Martin, 2020; Ziegler et al., 2020; Lv et al., 2021) and the highly virulent H5N1 influenza A virus (Baskin et al., 2009; Channappanavar et al., 2016), M1 macrophages have been associated with a high type I interferon response and cytokine storm leading to increased disease severity and mortality (Jamilloux et al., 2020). CD169+ tissue macrophages from patients who died from SARS-CoV-2 infection have been shown to express the SARS-CoV-2 entry receptor ACE2 and contain SARS-CoV-2 nucleoprotein (NP) (Feng et al., 2020; Jalloh et al., 2022). Although early studies showed that resident macrophages in human tissues do not express ACE2 (Sungnak et al., 2020; Zhao et al., 2020), more recent studies have shown that a specific subset of peripheral CD14+ monocytes and macrophages of healthy individuals express ACE2 (Zhang et al., 2021). In addition, C-type lectin receptors DC-SIGN (CD209), L-SIGN, and antibody-mediated internalization can provide further routes for viral entry of SARS-CoV and SARS-CoV-2 via endocytosis (Yang et al., 2004; Han et al., 2007; Xu et al., 2020; Amraei et al., 2021). Therefore, the decrease in the phagocytosis and phagocytic markers such as CD209 may help to alleviate the disease symptoms. When combined with our findings, this suggest that *KKC* may be a potential immunomodulatory agent against viral infections.

In conclusion, we report the impact of the medicinal polyherbal formulation *KKC* extract on polarization and phagocytic activity of primary human macrophages. Altogether, our data suggest that *KKC* extract modulates macrophage inflammatory response and could be a potential supplement for the treatment of infectious and inflammatory diseases. To the best of our knowledge, this is the first study investigating the effect of the *KKC* extract on primary human macrophage polarization and our data provide useful information on the immunomodulatory properties of *KKC*.

## Figures and Tables

**Table:** The components of *KKC*.

Cp. #	Botanical Name	Local Name	Common Name	Part
1	*Zingiber officinale* Roscoe	Chukku	Ginger	Rhizome
2	*Piper longum* L.	Thippili / Pippali	Long pepper	Fruit
3	*Syzygium aromaticum* (L.) Merr. & L.M.Perry	Kirambu	Clove	Flower bud
4	*Tragia involucrate* L.	Sirukanchori	Indian stinging nettle,climbing nettle	Root
5	*Anacyclus pyrethrum* (L.) Lag.	Akkirakaram	Pellitory	Root
6	*Hygrophila auriculata* (Schumach.) Heine	Mulliver	Vajradanti	Root
7	*Terminalia chebula* var.	Kadukkaithol	Yellow myroblan	Pericarp
8	*Adhatoda vasica* L.	Adathodai / Vasaka	Adathodai	Leaf
9	*Coleus amboinicus* Lour	Karpuravalli	Bishop’s weed	Leaf
10	*Saussurea lappa* (Decne.) Sch. Bip.	Kostam	Spiral Ginger	Root
11	*Tinospora cordifolia* (Willd.) Miers	Guduchi	Heart-leaved moonseed	Stem
12	*Clerodendron serratum* (L.) Moon	Siruthekku / Bhrangi	Beetle killer	Root
13	*Andrographis paniculata* (Burm.f.) Nees	Nilavmebu / Bhunimba	Andrographis	Whole plant
14	*Sida acuta* Burm.f.	Vattatthiruppi	Morning mallow	Root
15	*Cyperus rotundus* L.	Korai kizhangu	Cocgrass	Rhizome
